# Spatial variation in the morphological traits of *Pocillopora verrucosa* along a depth gradient in Taiwan

**DOI:** 10.1371/journal.pone.0202586

**Published:** 2018-08-17

**Authors:** Derek Soto, Stephane De Palmas, Ming Jay Ho, Vianney Denis, Chaolun Allen Chen

**Affiliations:** 1 Biodiversity Program, Taiwan International Graduate Program, Academia Sinica and National Taiwan Normal University, Taipei, Taiwan; 2 Department of Life Science, National Taiwan Normal University, Taipei, Taiwan; 3 Biodiversity Research Center, Academia Sinica, Taipei, Taiwan; 4 Green Island Marine Research Station, Academia Sinica, Ludao, Taiwan; 5 Institute of Oceanography, National Taiwan University, Taipei, Taiwan; Pennsylvania State University, UNITED STATES

## Abstract

*Pocillopora verrucosa* is a widely distributed depth-generalist coral that presents plasticity in its skeletal macro- and microstructure in response to environmental gradients. Light and water movement, which covary with depth, are the main environmental drivers of morphological plasticity in this genus; however, assessing environmentally-induced plasticity may be confounded by the extent of interspecific variation in *Pocillopora*. We examine the morphology of 8 typed *P*. *verrucosa* specimens collected along a depth gradient ranging from 7 to 45 meters and comprising 3 sites throughout Ludao, Taiwan. We measured 36 morphological characters, 14 which are novel, in 3 regions on the corallum—the apex, branch and base—in order to quantify their relationship to site and depth. We found significant correlation between depth and 19 morphological characters, notably branch verruca area, branch verruca height, base verruca spacing, base spinule length, and branch corallite area. 60% of microstructural characters and 25% of macrostructural characters showed a correlative relation to depth, suggesting that depth acclimatization is manifested primarily at the microstructural level. Canonical discriminant analysis of all morphometric characters by depth supports clustering into 3 groups: an overlapping 7m and 15m group, a 23-30m group, and a 38-45m group. Canonical discriminant analysis by site supports clustering into low- and high-current sites, differentiated primarily by branch septa width, base septa width, pre-terminal branch width, terminal branch maximum length, and terminal branch minimum length. We conclude that distinctive patterns of morphological variation in mesophotic specimens of *P*. *verrucosa* could reflect the effects of abiotic parameters such as light and water flow. Elucidating the mechanisms behind the morphological changes that occur in response to environmental gradients can help clarify the role that physiological plasticity plays in the acclimatization of corals to the unique environmental settings of mesophotic coral ecosystems.

## Introduction

Scleractinian coral taxonomy is traditionally based on descriptions of skeletal micro- and macrostructures, yet these characteristics can be altered in novel environments or in response to environmental change [1] through a phenomenon known as plasticity [2–5]. Morphological plasticity, which often transcends species boundaries, is a source of considerable confusion in the identification and taxonomy of many scleractinian corals [4, 6, 7] and may lead to the inclusion of misidentified species in a study [8]. In this context, the identification of stable morphological characters, synthesized with molecular taxonomy, is becoming increasingly important to an ongoing effort to strengthen species delineations in corals [9,10]. Despite its relevance to taxonomy and coral ecology, studies on morphological plasticity in corals—particularly along environmental gradients—are uncommon; out of more than 1300 extant scleractinian species, relatively few have been investigated for plastic responses [6].

The environmental setting of mesophotic coral ecosystems (MCEs) is characterized primarily by reduced light availability as a function of depth [11,12]. Light availability, along with water movement, are the main environmental parameters that influence coral morphology [6,13]; yet these parameters may be significantly attenuated at mesophotic depths [14]. In comparison to corals from shallow waters, corals that inhabit mesophotic environments may possess unique characteristics that optimize light harvesting and photosynthetic efficiency [15–17] and reduce oxidative stress [18, 19]. Traits exhibited by depth-specialized corals include: platy morphology [20], skeletal structures that increase how much light travels through coral tissue [21,22], unique microbiomes [23], increased photosynthetic pigments [12], monolayered symbiont cell arrangements [24], and *Symbiodinium* clade specificity [25,26]. Similarly, coral species with broad bathymetric distributions, commonly termed depth-generalists, may exhibit morphological responses to depth gradients [27]. A change in phenotype in response to environmental variation and achieved through non-genetic modification is termed acclimatization [28]. For example, branching corals such as Red Sea *Stylophora pistillata* present flatter morphologies and thinner branches in mesophotic environments [29]. Shade-adjusted colonies of *Montipora monasteriata* photoacclimate by means of growth patterns that preferentially expand surface area instead of volume [30]. Flatter colony morphologies occurring in deep water require less biomass, which in turn reduces photosynthetic demand [31]. At the microstructural scale, high calical relief in shallow *Orbicella annularis* (*ex*. *Montastraea annularis*) provides shading effects as a protective adaptation against UV radiation, while flatter calices in deeper morphs may serve to increase exposure to incident light [32]. Even though the interaction between light and depth on coral traits is well documented [32–38], the acclimatory potential of zoxanthellate scleractinian corals inhabiting MCEs remains poorly understood [39–41].

Corals within the genus *Pocillopora* possess a broad geographical range—spanning the tropical Pacific Ocean, Indian Ocean, and Red Sea—and comprises tropical to temperate latitudes and shallow to mesophotic settings [42,43]. Concomitant with this ample range is exposure to heterogenous habitats and environmental conditions, which include marginal environments [44,45]. Interspecific variation in morphological traits is frequently reported in *Pocillopora*, but it is often difficult to distinguish from intraspecific morphological plasticity [46–53].

*Pocillopora verrucosa* is a submassive branching coral common in shallow-water [54] and some mesophotic habitats [55,56] throughout the tropical Indo-Pacific, Eastern Pacific and Red Sea. *P*. *verrucosa* is described as possessing calices ranging 0.5–1.3mm in diameter, 2 cycles of irregularly sized and spaced septa, verrucae ranging 3-7mm in diameter and 2-6mm in height, and minimum branch diameter that typically does not exceed approximately twice the diameter of the smallest branch [49, 57]. *P*. *verrucosa* is highly plastic and presents developmental plasticity in larvae exposed to acidified environments [58], metabolic plasticity in response to temperature [59,60], physiologic plasticity, and symbiont specificity in response to depth and light gradients [61–63], and morphological plasticity in response to hydrodynamic flow patterns [64–67]. In the waters off of Ludao, Taiwan, *P*. *verrucosa* occupies a known vertical distribution ranging from 7-60m depth [68], making it ideal for studying the interaction between morphology and depth.

Here we examine how morphological traits in *P*. *verrucosa* relate to site and depth by measuring 36 characters in validated *P*. *verrucosa* specimens collected along a depth gradient ranging from shallow to upper mesophotic regions in Ludao, Taiwan. We then further examined the relationship between depth and morphological trait structure using a regressive analysis and a multivariate statistical approach. We postulate that *P*. *verrucosa* originating from mesophotic environments will present acclimatory changes in their morphology when compared to their shallow water counterparts.

## Materials & methods

**Fig 1:**
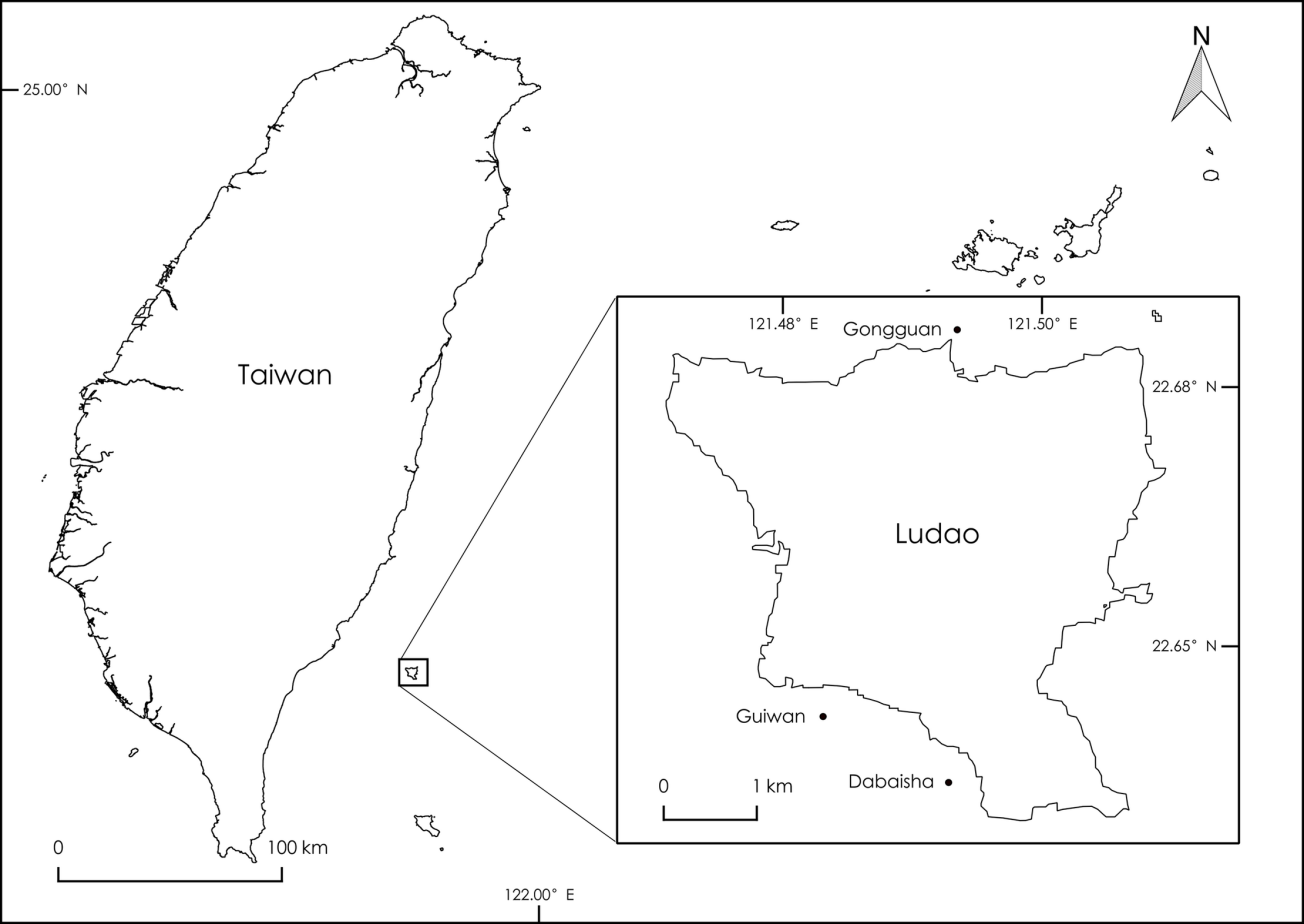
Study sites at Ludao, Taiwan. Samples were collected at 7m, 15m, 23-30m, and 38-45m depths at Guiwan, Dabaisha, and Gongguan reefs.

In order to characterize differences in morphology in response to depth, we measured 36 morphological characters in *Pocillopora verrucosa* collected from 4 depth profiles ranging from 7 to 45m. Samples were collected from 3 sites encompassing Ludao (also known as Green Island), an offshore volcanic island located off the southeast coast of Taiwan (Fig 1), during 4 field trips spanning 2016 to 2017. Coral fragments were collected by divers, using recreational and technical SCUBA diving, at four depth intervals—7m, 15m, 23-30m and 38-45m (± 1.25m tidal range)—and three sites—Guiwan (22.64022°N, 121.48113°E), Dabaisha (22.63678°N, 121.49026°E) and Gongguan (22.67750°N, 121.49415°E)—off the southern, southwestern and northern coasts of Ludao, respectively. Springtime marine environmental conditions in Guiwan measured by CTD, range between 24.5–25.2°C and 34.6–34.9 PSU salinity from the surface to 50m [68]. Light availability (PAR_surface_ = 1474 μmol (photons) m^2^/s) decreases exponentially from the surface to 3–8% total PAR at 50m [68]. Ludao interrupts the northeastward flow of the Kuroshio current and creates a wake off the island’s northeastern coast [69], consequently, current velocity at Dabaisha and Gongguan sites ranges between 0–0.5 m/s, while current at Guiwan ranges between 0.7–1.2 m/s [70].

**Fig 2:**
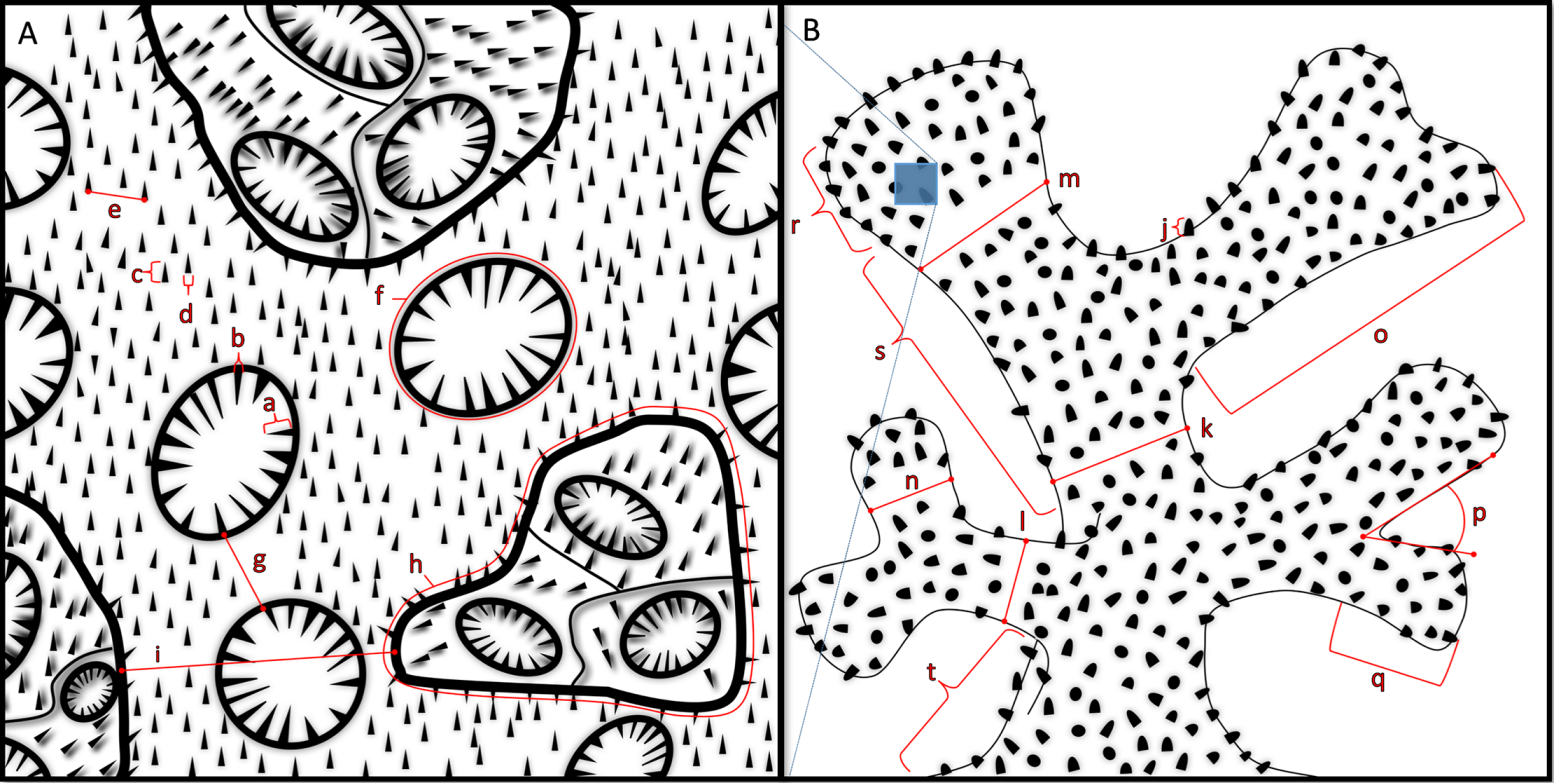
**Schematic of morphological traits and regions measured in this study at A. detailed and B. gross perspectives.** Features not drawn to scale. a. Septa Length b. Septa Width c. Spinule Length d. Spinule Width e. Spinule Spacing f. Corallite Area g. Corallite Spacing h. Verruca Area i. Verruca Spacing j. Verruca Height k. Pre-terminal Branch Maximum Width l. Pre-terminal Branch Minimum Width m. Terminal Branch Maximum Width n. Terminal Branch Minimum Width o. Terminal Branch Maximum Length p. Mean Branching Angle q. Terminal Branch Minimum Length r. Apex Region s. Branch Region t. Base Region.

**Fig 3:**
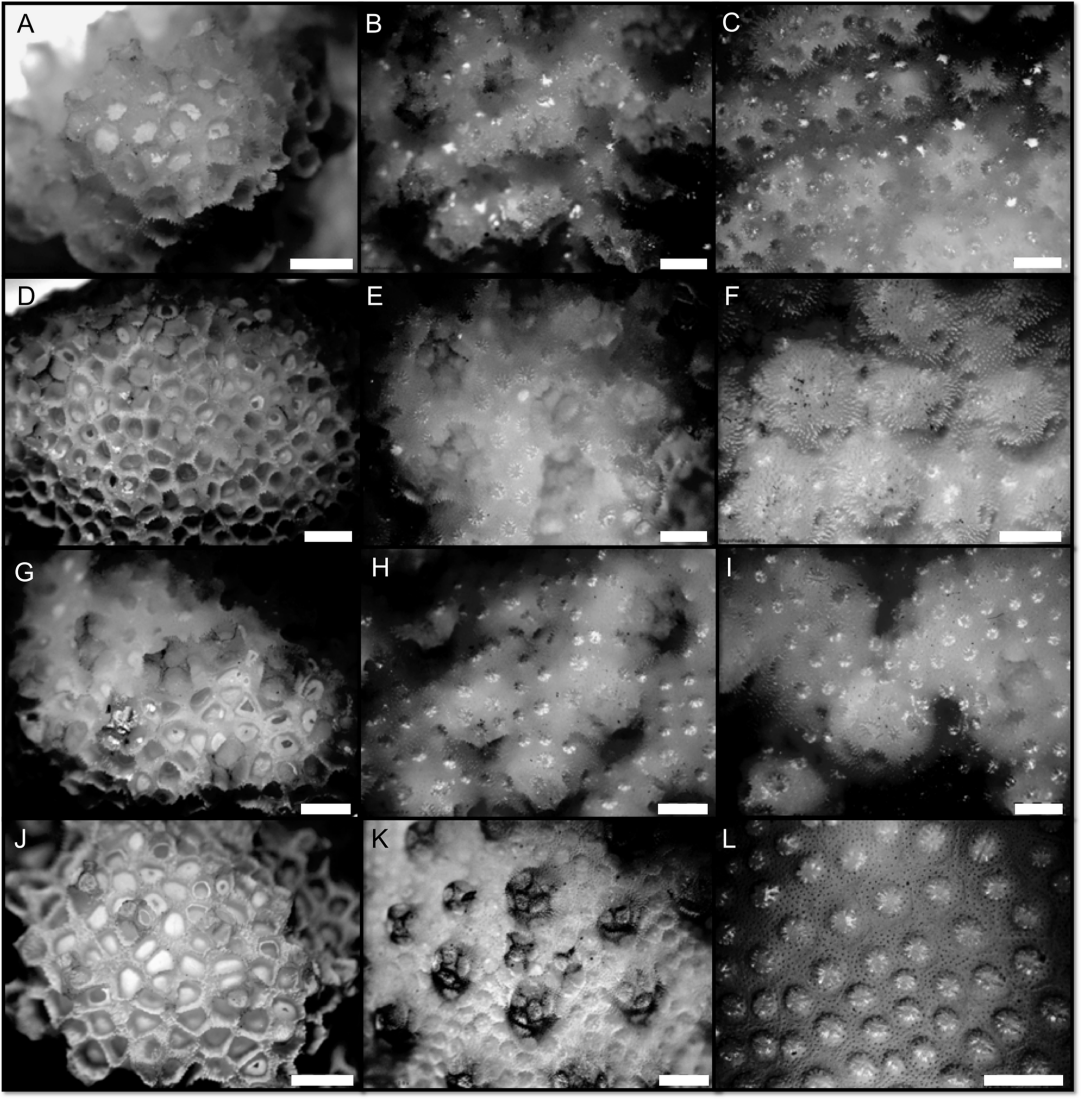
Skeletal structure of 3 distinct morphological regions (apex, branch, base) of *Pocillopora verrucosa* collected from 4 depth ranges (7m, 15m, 23-30m, 38-45m). A. 7m apex region B. 7m branch region C. 7m base region D. 15m apex region E. 15m branch region F. 15m base region G. 30m apex region H. 30m branch region I. 30m base region J. 45m apex region K. 45m branch region L. 45m base region. Scale bar equals 2 mm.

Colonies possessing *Pocillopora verrucosa*-like morphology were identified following [57]. Colony fragments were collected and transported to the lab where tissue sections were subsampled for molecular analyses. Skeletons were tagged, bleached, rinsed and dried for analysis of morphometric characters. Species identity for coral specimens was assessed through DNA sequencing of the open reading frame in the mitochondrial genome (*mtORF*) (detailed in de Palmas et al. 2018, *in preparation*). 85 total colonies were retained for this study (Table 1). Eight characters were measured at the gross colony level (macrostructural characters, hereafter) and 28 microstructural characters (Table 2) (Fig 2A and 2B) were measured within three distinct regions on the corallum designated as apex, branch, and base regions (microstructural characters, hereafter) (Fig 3). Independence of morphological characters was verified by checking a correlation plot for evidence of collinearity (S1 Fig). The apex region is delimited by the area at the tip of the branches (Fig 2B) and is distinguished by its large polygonal corallites lacking septa or columella and a narrow coenosteum containing broad, dentate spinules (Fig 3A, 3D, 3G and 3J). Verrucae in the apex are short and underdeveloped. The branch region is designated by the area on the branches proximal to the apex region (Fig 2B). This area is characterized by circular or oval plocoid corallites containing well-defined septa; columella may be present (Fig 3B, 3E, 3H and 3K). The coenosteum within the branches is broader than in the apical region and contains many rows of long thorn- or needle-shaped spinules. Verrucae in this region are columnar in shape, and abundant. The base is defined as the primary stem that forms the principal support to which all secondary branches of the corallum attach and that anchors the colony to the substrate (Fig 2B). The base region is distinguished by round or oval-shaped corallites that may or may not possess internal structure and a relatively extensive coenosteum that contains abundant rows of spinules (Fig 3C, 3F, 3I and 3L). Characters were digitally measured using an Olympus SZ2-ILST microscope and an Olympus DP72 camera coupled with cellSens Standard software calibrated using an objective micrometer slide. Colony density was calculated by measuring dry and buoyant colony weight in deionized water applied to Archimedes’ Principle modified to calculate the density of an object [71].

**Table 1:** Samples collected by site and depth.

Site Depth	Guiwan	Dabaisha	Gongguan	Total
**7m**	3	5	3	11
**15m**	9	9	2	20
**23-30m**	9	6	7	22
**38-45m**	12	9	11	32
**Total**	33	29	23	85

**Table 2:** Morphological characters measured in this study, and references to studies on *Pocillopora* which incorporate quantitative assessments of each character.

Character	Description	Replicates per Colony	References
Apex Spinule Length	Maximum length of apical spinule	10	-
Apex Spinule Width	Maximum width of apical spinule	10	-
Apex Spinule Spacing	Minimum distance between adjacent apical spinules	10	-
Apex Corallite Area	Area demarcated by rim of apical corallite	10	[7]
Apex Corallite Spacing	Minimum distance between adjacent apical corallites.	10	[7]
Apex Verruca Area	Area demarcated by base of apical verruca	10	[7, 10]
Apex Verruca Spacing	Minimum distance between adjacent apical verruca	10	[7]
Apex Verruca Height	Length of apical verruca measured from base to tip	10	[7]
Base Spinule Length	Maximum length of base spinule	10	[72]
Base Spinule Width	Maximum width of base spinule	10	[72]
Base Spinule Spacing	Minimum distance between adjacent base spinules	10	-
Base Septa Width	Maximum width of basal primary corallite septa	10	-
Base Septa Length	Maximum length of basal primary corallite septa	10	-
Base Corallite Area	Area demarcated by rim of basal corallite	10	-
Base Corallite Spacing	Minimum distance between adjacent base corallites.	10	-
Base Verruca Area	Area demarcated by base of basal verruca	10	-
Base Verruca Spacing	Minimum distance between adjacent basal verruca	10	-
Base Verruca Height	Length of basal verruca measured from base to tip	10	-
Branch Spinule Length	Maximum length of branch spinule	10	[72]
Branch Spinule Width	Maximum width of branch spinule	10	[72]
Branch Spinule Spacing	Minimum distance between adjacent branch spinules	10	-
Branch Septa Width	Maximum width of branch primary corallite septa	10	-
Branch Septa Length	Maximum length of branch primary corallite septa	10	-
Branch Corallite Area	Area demarcated by rim of branch corallite	10	[7]
Branch Corallite Spacing	Minimum distance between adjacent branch corallites.	10	[7]
Branch Verruca Area	Area demarcated by base of branch verruca	10	[7]
Branch Verruca Spacing	Minimum distance between adjacent branch verruca	10	[7,10]
Branch Verruca Height	Length of branch verruca measured from base to tip	10	[7]
Colony Density	Colony density calculated using Archimedes’ Principle.	1	[73]
Terminal Branch Maximum Width	Maximum diameter measured halfway between the branch tip and the branching point of the ultimate ramification	1	[7, 10, 67, 74, 75]
Terminal Branch Minimum Width	Minimum diameter measured halfway between the branch tip and the branching point of ultimate ramification	1	[7, 10, 67, 74, 75]
Pre-terminal Branch Maximum Width	Maximum diameter measured halfway between the branching point of ultimate ramification and branching point of penultimate ramification	1	[7, 10]
Pre-terminal Branch Minimum Width	Minimum diameter measured halfway between the branching point of ultimate ramification and branching point of penultimate ramification	1	[7, 10]
Terminal Branch Maximum Length	Length of longest ultimate branch measured as the distance between branch tip and branching point of ultimate ramification	1	[10]
Terminal Branch Minimum Length	Length of shortest ultimate branch measured as the distance between branch tip and branching point of ultimate ramification	1	[10]
Mean Branching Angle	Interior angle measured between adjacent ramifications	5–10	[7, 74]

Statistical tests were performed using R version 3.4.2 [76]. We estimated the relationship between morphological traits and depth using Kendall’s rank correlation coefficient from R package “stats”. Morphological characters were assessed for multicollinearity via a correlation matrix created using the R package “corrplot”. Pairwise characters possessing a Pearson’s r > 0.8 (n = 1) were considered collinear following [77] and one character was retained for discriminant analysis. Normality was assessed via the Shapiro-Wilk test and data were log-transformed in order to meet the assumptions of parametric tests. Multivariate homogeneity of variances was assessed using “vegan” package [78]. Canonical discriminant analyses were visualized using the R package “candisc” [79].

## Results

**Fig 4:**
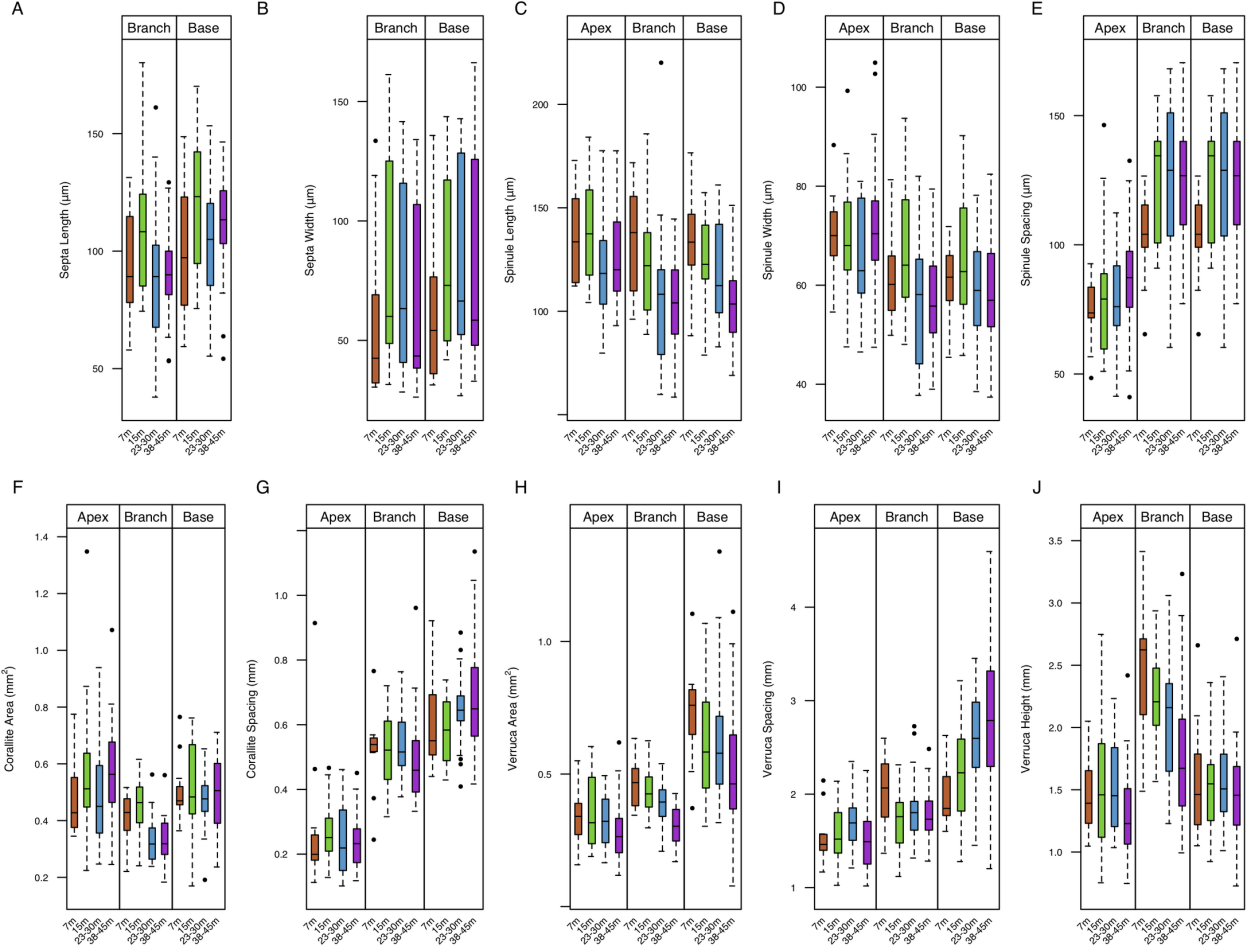
Variation in microstructural characters from the apex, branch, and base regions of the corallum in response to depth. A. Septa Length B. Septa Width C. Spinule Length D. Spinule Width E. Spinule Spacing F. Corallite Area G. Corallite Spacing H. Verruca Area I. Verruca Spacing J. Verruca Height. Boxes indicate median ± quartiles. Whiskers indicate 5^th^ and 95^th^ percentiles, respectively.

**Fig 5:**
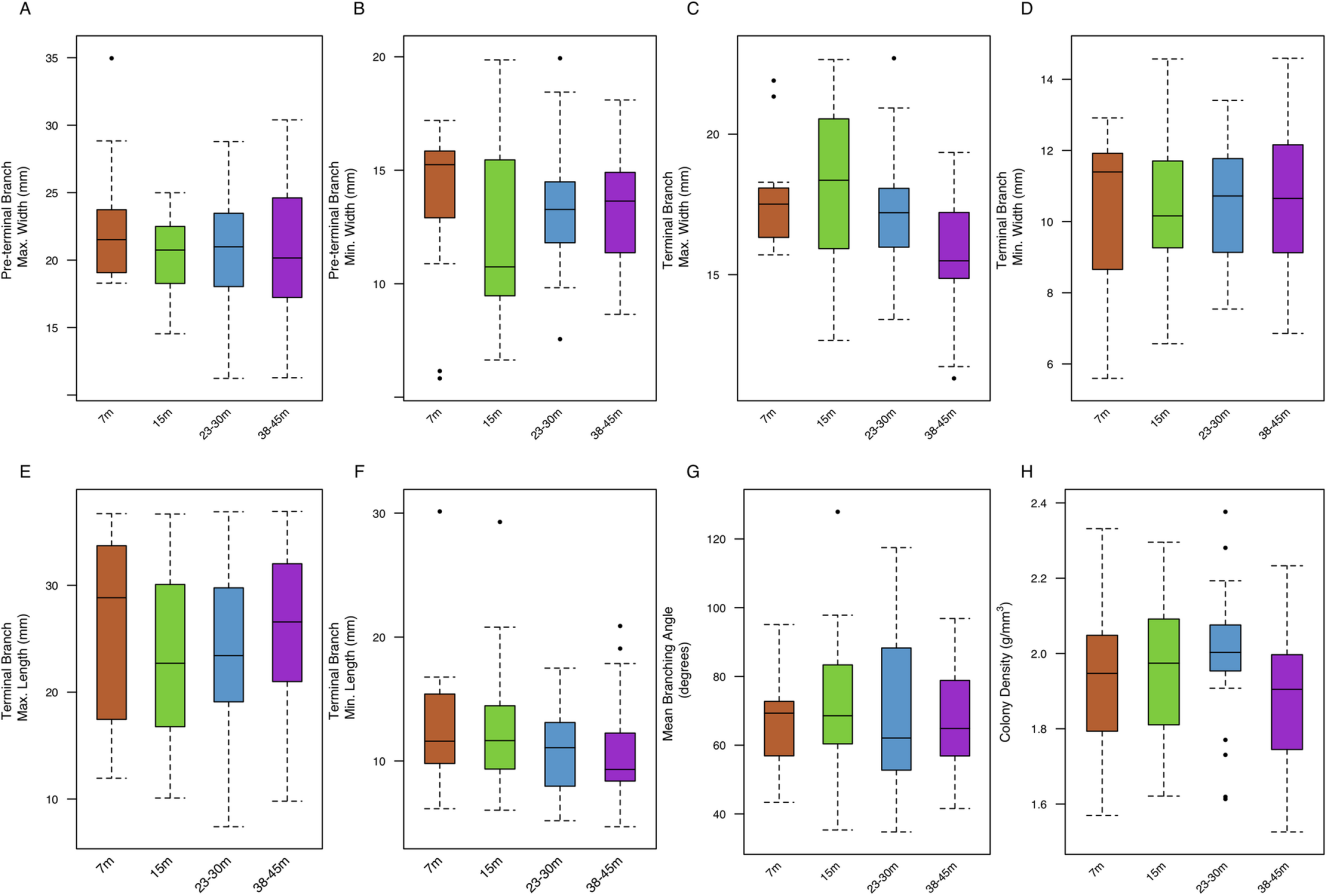
Variation in macrostructural characters of *P*. *verrucosa* in response to depth. A. Pre-terminal Branch Maximum Width B. Pre-terminal Branch Minimum Width C. Terminal Branch Maximum Width D. Terminal Branch Minimum Width E. Terminal Branch Maximum Length F. Terminal Branch Minimum Length G. Branching Angle H. Colony Density. Boxes indicate median ± quartiles. Whiskers indicate 5^th^ and 95^th^ percentiles, respectively.

Out of 28 microstructural characters (Fig 4) and 8 structural characters (Fig 5) measured, significant correlation coefficients were found for 19 characters: apex spinule length, apex spinule spacing, apex verruca area, apex verruca height, base spinule length, base spinule width, base corallite area, base corallite spacing, base verruca area, base verruca spacing, branch spinule length, branch spinule width, branch spinule spacing, branch septa length, branch corallite area, branch verruca area, branch verruca height, terminal branch maximum width, and terminal branch minimum length (Table 3). However, significant correlation coefficients that explain greater than 30% of the observed variance were found for branch verruca area, (τ = -0.50), branch verruca height (τ = -0.39), base verruca spacing (τ = 0.37), base spinule length (τ = -0.35), and branch corallite area (τ = -0.30). Significant correlations to depth were found in eleven previously described characters (50%) and eight (57%) newly described characters. Trait distribution varies along a depth gradient (S2 Fig). The following characters showed no correlation to depth: apex spinule width, apex corallite area, apex corallite spacing, apex verruca spacing, base spinule spacing, base septa width, base septa length, base verruca height, branch septa width, branch corallite spacing, branch verruca spacing, colony density, terminal branch minimum width, pre-terminal branch maximum width, pre-terminal branch minimum width, terminal branch maximum length, and mean branching angle.

**Table 3:** Correlation between morphological characters and depth. Significant characters are presented in boldface.

Trait	Abbreviation	Kendall’s τ	z	p-value
Apex Spinule Length	20	-0.17	-2.1	**0.033**
Apex Spinule Width	23	0.042	0.50	0.61
Apex Spinule Spacing	26	0.27	3.27	**0.0010**
Apex Corallite Area	1	0.010	0.12	0.89
Apex Corallite Spacing	2	-0.10	-1.23	0.21
Apex Verruca Area	3	-0.22	-2.71	**0.0065**
Apex Verruca Spacing	4	-0.061	-0.74	0.45
Apex Verruca Height	5	-0.17	-2.08	**0.037**
Base Spinule Length	22	-0.35	-4.29	**1.7e-05**
Base Spinule Width	25	-0.22	-2.68	**0.0071**
Base Spinule Spacing	28	0.10	1.26	0.20
Base Septa Width	18	0.025	0.31	0.75
Base Septa Length	19	-0.070	-0.85	0.39
Base Corallite Area	6	0.22	0.82	**0.018**
Base Corallite Spacing	7	0.23	2.85	**0.0043**
Base Verruca Area	8	-0.26	-3.23	**0.0012**
Base Verruca Spacing	9	0.37	4.56	**4.99e-06**
Base Verruca Height	10	-0.047	-0.57	0.56
Branch Spinule Length	21	-0.26	-3.21	**0.0013**
Branch Spinule Width	24	-0.20	-2.52	**0.011**
Branch Spinule Spacing	27	0.19	2.37	**0.017**
Branch Septa Width	16	-0.087	-1.05	0.29
Branch Septa Length	17	-0.23	-2.79	**0.0052**
Branch Corallite Area	11	-0.30	-3.69	**0.00022**
Branch Corallite Spacing	12	-0.14	1.81	0.069
Branch Verruca Area	13	-0.50	-6.16	**7.1e-10**
Branch Verruca Spacing	14	-0.14	-1.78	0.073
Branch Verruca Height	15	-0.39	-4.81	**1.4e-06**
Colony Density	29	-0.13	-1.61	0.10
Terminal Branch Maximum Width	30	-0.26	-3.18	**0.0014**
Terminal Branch Minimum Width	31	0.028	0.0023	0.97
Pre-terminal Branch Maximum Width	32	-0.067	-0.81	0.41
Pre-terminal Branch Minimum Width	33	-0.018	-0.22	0.82
Terminal Branch Maximum Length	34	-0.037	-0.45	0.65
Terminal Branch Minimum Length	35	-0.22	-2.73	**0.0062**
Mean Branching Angle	36	0.041	0.49	0.62

**Fig 6:**
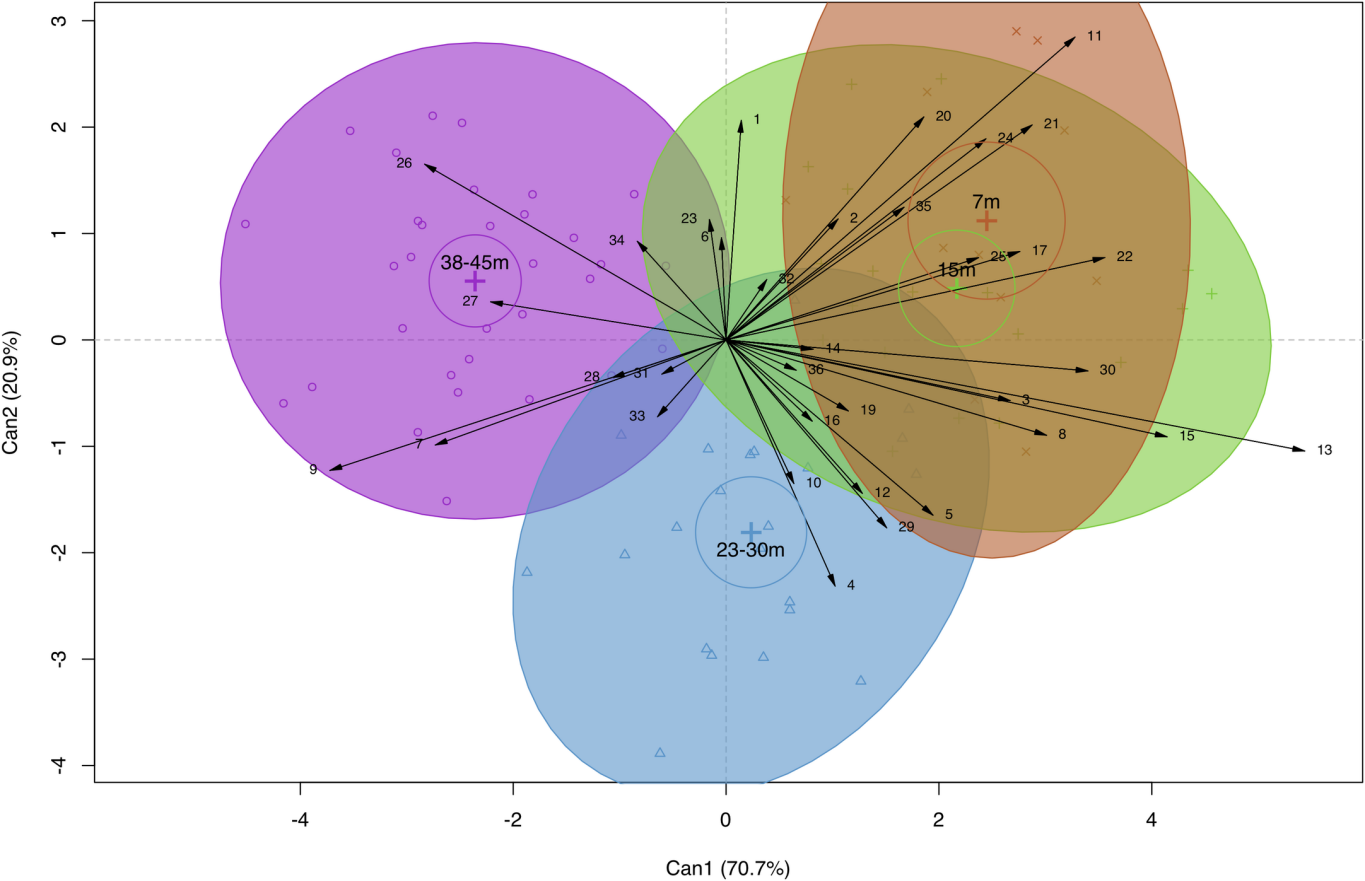
Canonical discriminant analysis of mean structural and microstructural character means in *P*. *verrucosa* sorted by depth. Plot of first two canonical variables (Can1 vs. Can2) with loadings in parentheses. Crosses represent group centroids, circles surrounding crosses represent 95% confidence coefficient for group centroids, ellipses represent 95% coverage probability. Biplot displays the magnitude and direction of each character’s effect on group discrimination. Highly collinear characters are excluded. Vector abbreviations follow Table 3.

Canonical discriminant analysis of all morphological characters by depth (Fig 6) shows a high degree of overlap in canonical space for 7m and 15m groups. All groups partially overlap each other to some degree, except for the 7m and 38-45m groups, which do not intersect at all. First and second canonical variates account for 91.6% of observed among-group variance. Only the first canonical variate explains the observed differentiation between depth groups (LR_C1_ = 0.057, F_C1 105,141_ = 2.14, p<0.001; LR_C2_ = 0.29, F_C2 68,96_ = 1.17, p = 0.23). Differentiation between group centroids in canonical space by means of a permutation test for homogeneity of multivariate dispersions was non-significant (F_3,81_ = 1.33, p = 0.27) (Figure A in S3 Fig). Vectors 9, 11, 13, 15, 22—corresponding to base verruca spacing, branch corallite area, branch verruca area, branch verruca height and base spinule length, respectively—possessed noteworthy discriminatory potential.

**Fig 7:**
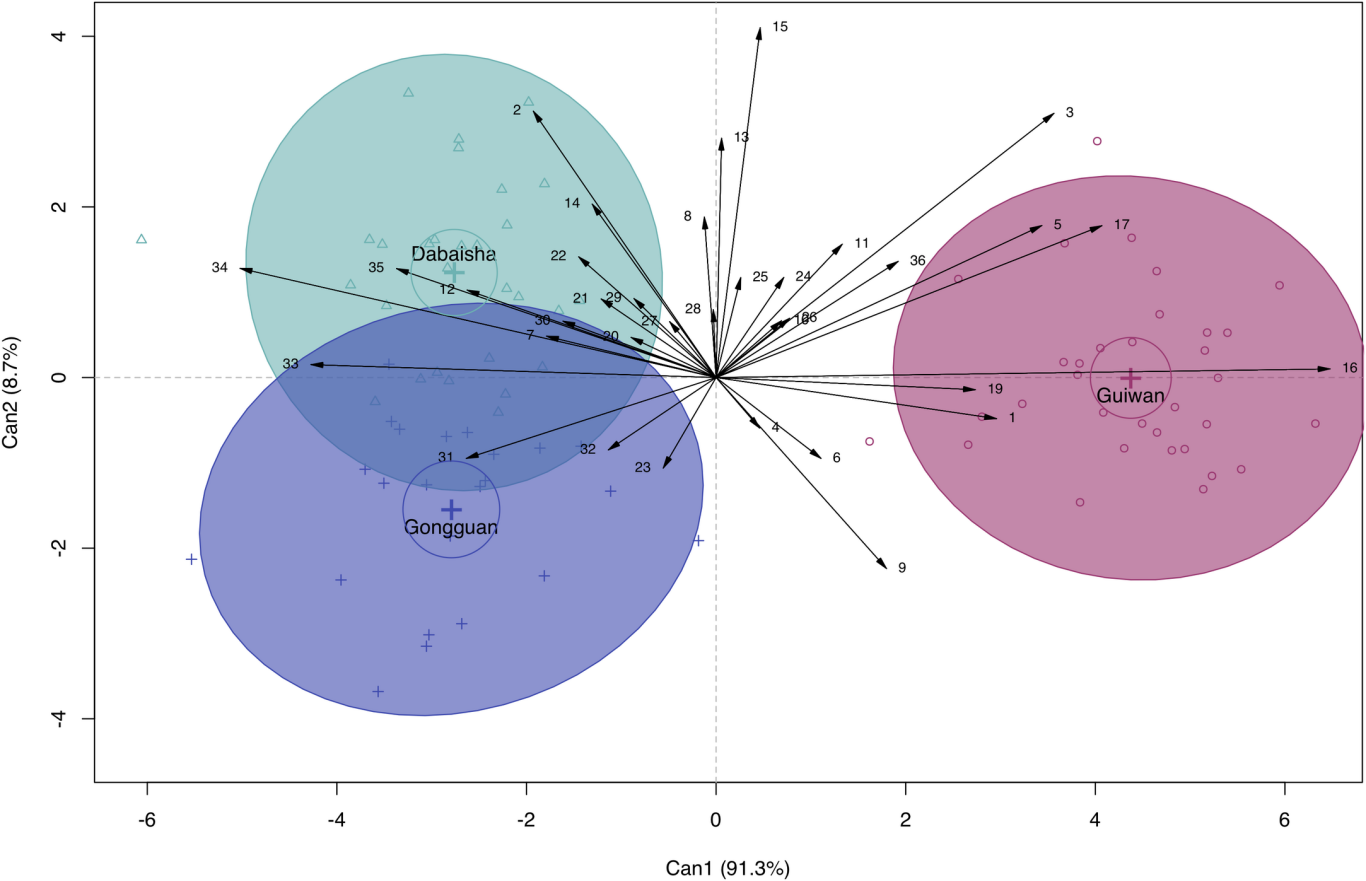
Canonical discriminant analysis of structural and microstructural character means of *P*. *verrucosa* sorted by site. Plot of first two canonical variables (Can1 vs. Can2) with loadings in parentheses. Crosses represent group centroids, circles surrounding crosses represent 95% confidence coefficient for group centroids, ellipses represent 95% coverage probability. Biplot displays the magnitude and direction of each character’s effect on group discrimination. Highly collinear characters are excluded. Vector abbreviations follow Table 3.

Canonical discriminant analysis of all characters by site (Fig 7) shows Guiwan uncoupled from the overlapping Dabaisha and Gongguan groups. First and second canonical variates account for 100% of observed among-group variance. Canonical correlations between the sites and the first and second canonical variates were significant (LR_C1_ = 0.03, F_70,96_ = 6.13, p<0.001; LR_C2_ = 0.45, F_34,49_ = 1.73, p = 0.03). A permutation test for homogeneity of multivariate dispersions does not show significant differentiation between group centroids (F_2,82_ = 0.80, p = 0.45) (Figure B in S3 Fig). Discriminant vectors by site include 3, 16, 17, 33, 34, 35 which correspond to apex verruca area, branch septa width, branch septa length, base septa width, pre-terminal branch minimum width, terminal branch maximum length, and terminal branch minimum length, respectively.

## Discussion

Here we demonstrate variation in 17 micro- and 2 macrostructural characters, 8 of which are novel, in colonies of *Pocillopora verrucosa* occurring along a depth gradient. This study is among a few that examine variation in morphological traits within a mesophotic setting (e.g. [80]). Our results show substantial overlap between 7 and 15m groups in canonical space, suggesting that these groups are morphologically indistinguishable (Fig 6). In contrast, there is no overlap between the 7 and 38-45m groups, which is indicative of strong morphological divergence. Limited separation between groups implies that morphological variation has a gradational nature, while partial intersection between groups suggests that some characters are invariant in response to depth. Because we were unable to determine whether group discrimination originates from among-group dispersion or from actual variation in distance to centroid, interpretation of these results warrants caution (Figure A in S3 Fig). Character vectors possessing highest discriminatory potential (Fig 6) were coincident with characters possessing highest correlation coefficients (Table 3). 60% of microstructural characters and 25% of macrostructural characters exhibited significant variation with depth, suggesting that acclimatization to depth is manifested primarily at the microstructural level. Canonical discrimination between sites (Fig 7) reveals that colonies from Guiwan—an exposed, high water velocity site—are morphologically distinct from those originating from Dabaisha and Gongguan—which are sheltered, low-velocity sites [70]. Site discrimination is principally driven by branch and septal characters, which indicates that differences between site manifest at both the micro- and macrostructural level.

We found that increases in depth correspond to shorter spinules in all regions, narrower spinules in the base and branch regions, and increases in spinule spacing in the apex and branch regions. To the best of our knowledge, no studies have reported changes in spinule morphology in relation to depth. However, specimens of *Pocillopora damicornis* exposed to high wave-action presented reductions in the length and width of skeletal spines (spinules) [72], indicating that spinule morphology is sensitive to environmental gradients. Skeletal spinules play a role in the adhesion of the tissue-skeleton interface [72]. Yet, in this context, we believe the variation in spinule morphology is best explained by variation in growth and calcification rates with depth [81].

Our results do not support significant differentiation between base and branch septa width and base septa length with depth, yet we did observe significant reductions in branch septa length in our specimens. In *Montastraea cavernosa*, decreased exposure to light can produce corallites with fewer septa [82]. Septal length and width have also been shown to increase with depth [34] however, this contrasts with a recent study where septa were significantly shortened in mesophotic *M*. *cavernosa* [80]. Cross-transplanted colonies of *Goniastrea pectinata* developed longer septa when transplanted to deeper stations to maximize light efficiency at low irradiances [83]. The effects of depth on septal characters in the current study are inconsistent with the literature. Surprisingly, branch septa width was a significant factor in site discrimination, suggesting that septa morphology may be more sensitive to other environmental parameters such as water velocity and flow.

Our data show that corallite area in the branch regions correlate negatively with depth, while corallite spacing increases in the base regions. Corallite size and inter-corallite spacing are traits that vary with depth, paralleling reductions in ambient light intensity [84–85]. In *Montastraea cavernosa*, decreased exposure to light can produce smaller corallites [82] and reductions in corallite spacing [34]. Polyp living space (corallite spacing) increases with depth in *Dipsastraea pallida* (*ex*. *Favia pallida*) [86]. Modelling studies based on morphometric data in *Galaxea fascicularis* demonstrate that corallite width and inter-corallite distance decreased while corallite height and density increased when exposed to reductions in incident light associated with depth [87]. Smaller calyx diameters produce smaller angles of inclination within the calyx walls, which could increase shading; conversely, larger corallites allow light collection from greater angles, resulting in greater light capture [83]. Here, corallite size appears to vary non-linearly with depth, which could indicate shifts in the relative reliance of depth-generalist corals on autotrophy and heterotrophy at depth [88–89].

We determined that as depth increases, verruca size diminishes in all regions and verrucae become shorter within the apex and branch regions. Correspondingly, colonies of *Montipora capitata* produced more tuberculae, protrusions similar to verrucae in *P*. *verrucosa*, in high-light environments [90]. We postulate that taller and larger verrucae may provide protective shading in high-light environments while smaller and shorter verrucae may reduce self-shading, and increase light capture, in deeper environments. Our data show that verruca spacing increases with depth in the base region of the corallum, resulting in a relatively smooth surface. Colonies in deep water may preferentially adopt flatter colony morphologies to enhance exposure to incident light [91–92], which creates a shaded region on the underside of the colony. We postulate that this effect could be an acclimatory response to a low-light microenvironment [93–95] and provides evidence for the role of verrucae as photo-protective structures.

We did not observe any significant change in colony density with depth. This contrasts with other studies, where decreased exposure to light reduces calcification rates [96], and linear growth rates [86] resulting in increased colony density with depth [81]. In [73], comparisons of colony densities of 9 corals, showed reduced colony density with depth for most species, however, as in our study, results for *P*. *verrucosa* were inconclusive. We report negative correlations between terminal branch maximum width and terminal branch minimum length with depth, a result that parallels other studies in the literature. Branch thickness decreases in *Stylophora pistillata* occurring along a depth gradient ranging down to 65m [29]. Branch spacing and branch tip diameter increased and decreased, respectively, in colonies of *Madracis mirabilis* along a depth gradient [13]. Branch spacing was larger and branch length was shorter in *Acropora humilis* colonies from deep habitats compared to shallow-water conspecifics [37]. These changes in branch structure could stem from reduced growth rates with depth; however, branch characters were found to be important for site discrimination (Fig 7). Because branch morphology is strongly responsive to water flow [64–68], we propose that differential water velocities among sites could better explain differences in branch morphology.

Plasticity enables a species to occupy a range of microhabitats within the reef and may facilitate adaptation tailored to specific environmental conditions [5,6]. Irradiance and hydrodynamics are environmental traits known to exert the greatest effect over plasticity in corals [6,13]; however, sedimentation [97], nutrient availability [98], turbidity [99–100], and inter-species interactions [101] have also been shown to alter colony morphology. It is possible that water flow may preferentially exert selective influence over macrostructural characters such as branch spacing, diameter and thickness [13, 67, 74, 102], while light availability may be influential at both macrostructural and microstructural levels [37, 103–105]. Locally, light availability (PAR) exhibits substantial reduction along a vertical gradient [68]. Water velocity at 50m is also attenuated compared to 10m [70]. Little variation was observed in other environmental variables (temperature, salinity) for which data exist. Data on local sedimentation rates and nutrient availability, which may covary along depth profiles, are unavailable; thus we cannot not infer their roles within the context of this study. Fine-scale long-term monitoring of environmental parameters could be more conclusive. Nevertheless, we hypothesize that light and current are major environmental drivers behind morphological variation observed in our study.

Morphological traits often overlap in *Pocillopora* [52], blurring obvious transitions between species [10]. The confounded taxonomy of the genus is particularly encumbering in the field, where estimates of diversity require visual identification based on morphology and are impractical to achieve with genetic methods. Thus, identifying diagnostic morphological characters is essential for morphological species discrimination [10]; yet no studies have identified stable traits in the context of environmentally-induced plasticity. Here we identify various characters that exhibit little plasticity along a depth gradient. Detailed comparisons of morphology within and among other *Pocillopora* species, and done within the context of environmental plasticity, could help assess their utility as taxonomic markers. Avoiding species misidentification in *Pocillopora* is a real challenge because misidentified specimens can bias results (as discussed in [106]).

Morphological plasticity can occur in response to environmental conditions (phenotypic plasticity), as a product of genetic differentiation, or as a combination of both [6]. The relative contribution of environment and genetics to morphological plasticity can be species-dependent [107]: some species, such as *Stylophora pistillata*, are known to display extensive plasticity in response to environmental conditions [108, 109]; yet others, like *Pavona cactus*, are phenotypically stable and show little or no response to environmental variation [110].

Challenges in the taxonomy *of Pocillopora* underscore the relevance of implementing high-resolution morphometric methods and highlight the importance of integrating morphological and molecular approaches. To better estimate the role plasticity plays in determining the vulnerability of species to climate change [111], transplantation of clonal replicates over a range of environments [112, 113] can untangle sources of plasticity (environmental vs. fixed factors). Integration of omics approaches can further clarify the mechanisms behind the transgenerational transfer of plastic responses [28, 114] and should be considered in future studies.

## Supporting information

S1 Fig Correlation matrix of all morphological characters for verification of non-collinearity. Characters are numbered according to Table 3 and ordered by decreasing first principal component. Area and color of pies denotes absolute value of pairwise Pearson’s correlation coefficients. Red and blue hues represent positive and negative correlations, respectively. Only significant correlations are shown.
(TIFF)

S2 Fig Scaled kernel density plots of discriminant morphological characters by depth. Distributions are coded by depth. Red: 7m, green: 15m, blue: 23-30m, and purple: 38-45m.
(TIFF)

S3 Fig Distance to centroid in multivariate space. A. By depth B. By site. Boxes indicate median ± quartiles. Whiskers indicate 5^th^ and 95^th^ percentiles, respectively.
(TIFF)

S1 Dataset Spreadsheet containing raw data. (XLSX)
