# Supplementary figures and images for: Spatial variation in the morphological traits of Pocillopora verrucosa along a depth gradient in Taiwan
Source: PLoS One. 2018 Aug 17;13(8):e0202586. doi: 10.1371/journal.pone.0202586 (PMC6097691; doi:10.1371/journal.pone.0202586)

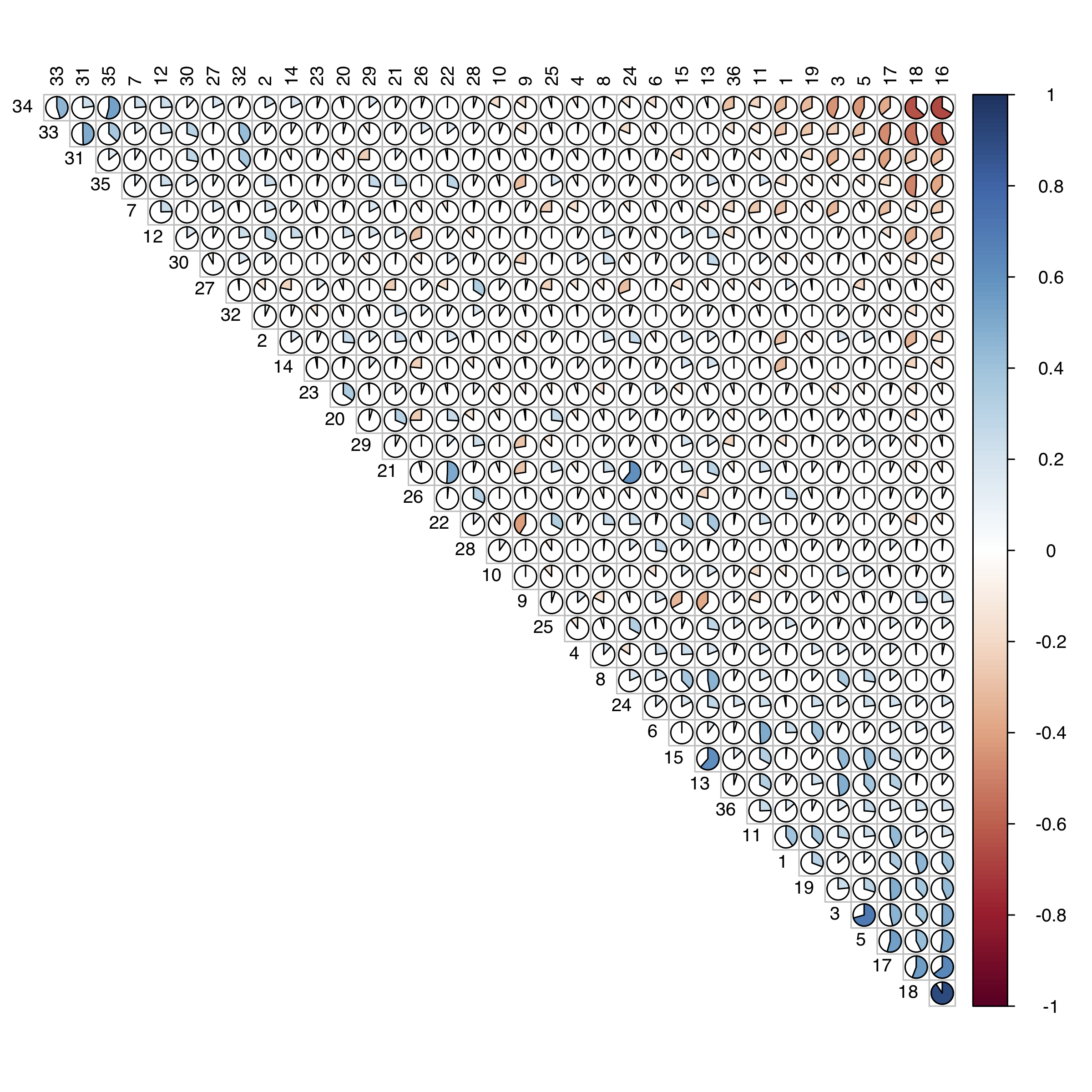

Supplement: S1 Fig — Characters are numbered according to Table 3 and ordered by decreasing first principal component. Area and color of pies denotes absolute value of pairwise Pearson’s correlation coefficients. Red and blue hues represent positive and negative correlations, respectively. Only significant correlations are shown. (TIFF) [file pone.0202586.s001.tiff]

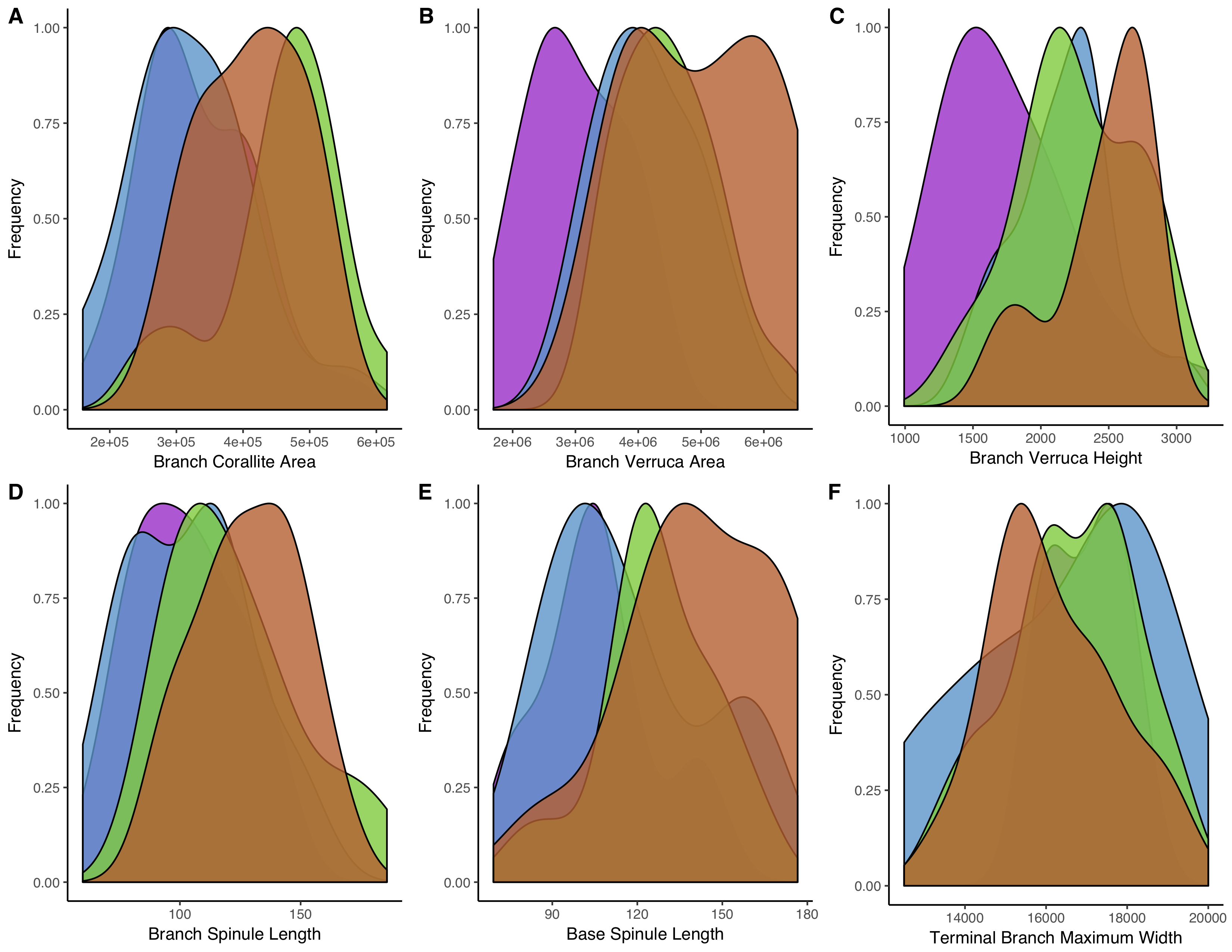

Supplement: S2 Fig — Distributions are coded by depth. Red: 7m, green: 15m, blue: 23-30m, and purple: 38-45m. (TIFF) [file pone.0202586.s002.tiff]

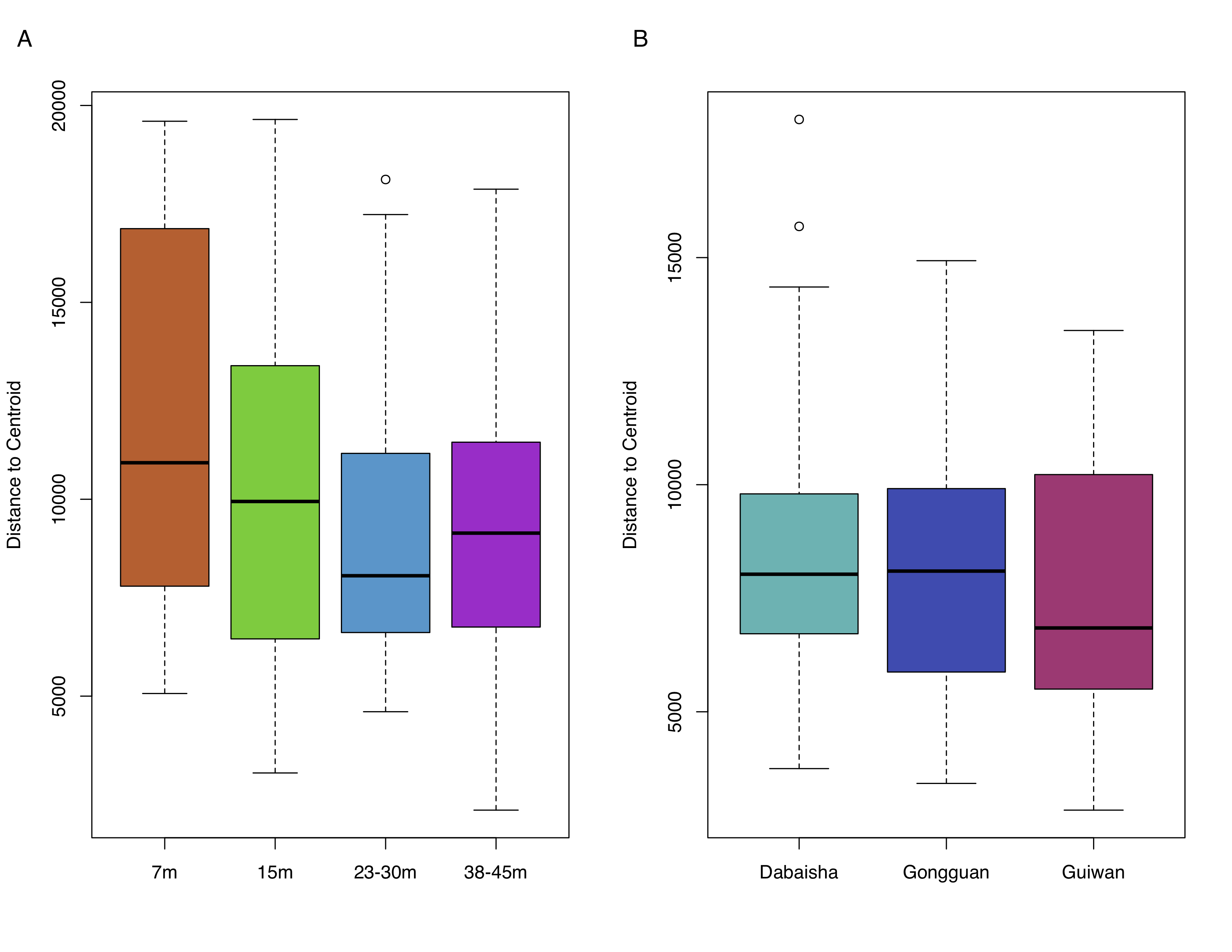

Supplement: S3 Fig — A. By depth B. By site. Boxes indicate median ± quartiles. Whiskers indicate 5th and 95th percentiles, respectively. (TIFF) [file pone.0202586.s003.tiff]
